# Influence of the Neuroprotective Properties of Quercetin on Regeneration and Functional Recovery of the Nervous System

**DOI:** 10.3390/antiox12010149

**Published:** 2023-01-07

**Authors:** Simone Ortiz Moura Fideles, Adriana de Cássia Ortiz, Daniela Vieira Buchaim, Eliana de Souza Bastos Mazuqueli Pereira, Maria Júlia Bento Martins Parreira, Jéssica de Oliveira Rossi, Marcelo Rodrigues da Cunha, Alexandre Teixeira de Souza, Wendel Cleber Soares, Rogerio Leone Buchaim

**Affiliations:** 1 Department of Biological Sciences, Bauru School of Dentistry (FOB/USP), University of Sao Paulo, Bauru 17012-901, Brazil; 2 Postgraduate Program in Structural and Functional Interactions in Rehabilitation, University of Marilia (UNIMAR), Marília 17525-902, Brazil; 3 Teaching and Research Coordination of the Medical School, University Center of Adamantina (UNIFAI), Adamantina 17800-000, Brazil; 4 Graduate Program in Anatomy of Domestic and Wild Animals, Faculty of Veterinary Medicine and Animal Science, University of Sao Paulo, Sao Paulo 05508-270, Brazil; 5 Medical Bill Audit, Holy House of Mercy (Santa Casa de Misericórdia), Marília 17515-900, Brazil; 6 Anatomy Department, Padre Anchieta University Center (UniAnchieta), Jundiai 13210-795, Brazil; 7 Department of Morphology and Pathology, Jundiaí Medical School, Jundiai 13202-550, Brazil; 8 Department of Medicine, University Center of Adamantina (UNIFAI), Adamantina 17800-000, Brazil; 9 Department of Exact Sciences, University Center of Adamantina (UNIFAI), Adamantina 17800-000, Brazil

**Keywords:** quercetin, nerve regeneration, nervous system, spinal cord, peripheral nerves

## Abstract

Quercetin is a dietary flavonoid present in vegetables, fruits, and beverages, such as onions, apples, broccoli, berries, citrus fruits, tea, and red wine. Flavonoids have antioxidant and anti-inflammatory effects, acting in the prevention of several diseases. Quercetin also has neuroprotective properties and may exert a beneficial effect on nervous tissue. In this literature review, we compiled in vivo studies that investigated the effect of quercetin on regeneration and functional recovery of the central and peripheral nervous system. In spinal cord injuries (SCI), quercetin administration favored axonal regeneration and recovery of locomotor capacity, significantly improving electrophysiological parameters. Quercetin reduced edema, neutrophil infiltration, cystic cavity formation, reactive oxygen species production, and pro-inflammatory cytokine synthesis, while favoring an increase in levels of anti-inflammatory cytokines, minimizing tissue damage in SCI models. In addition, the association of quercetin with mesenchymal stromal cells transplantation had a synergistic neuroprotective effect on spinal cord injury. Similarly, in sciatic nerve injuries, quercetin favored and accelerated sensory and motor recovery, reducing muscle atrophy. In these models, quercetin significantly inhibited oxidative stress and cell apoptosis, favoring Schwann cell proliferation and nerve fiber remyelination, thus promoting a significant increase in the number and diameter of myelinated fibers. Although there is still a lack of clinical research, in vivo studies have shown that quercetin contributed to the recovery of neurological functions, exerting a beneficial effect on the regeneration of the central and peripheral nervous system.

## 1. Introduction

Neurological disorders affect millions of people and can lead to complications that compromise quality of life [1,2]. Several factors may be associated with the etiology of neurological disorders, such as neurodegenerative and metabolic diseases, injuries, trauma, ischemia, and tumors. Among them, injuries and traumatic lesions have become a public health problem, considering the sequelae commonly associated with trauma [3]. Depending on the severity and the affected site, injury to the nervous system can lead to different degrees of functional disability and not always a satisfactory recovery can be achieved. Although nervous tissue has a certain regenerative capacity, several neurological functions can be impaired as a result of injuries to the central or peripheral nervous system. Spinal cord injuries can lead to partial or total loss of functional capacity [1,4]. Traumatic brain injury, in turn, is one of the main causes of death or disability and has been considered a risk factor for the development of neurodegenerative pathologies, such as Parkinson’s disease [5,6]. Similarly, peripheral nerve injuries can lead to neuropathies, sensory and motor deficits, muscle atrophy and neuritic pain [4,7,8].

**Figure 1:**
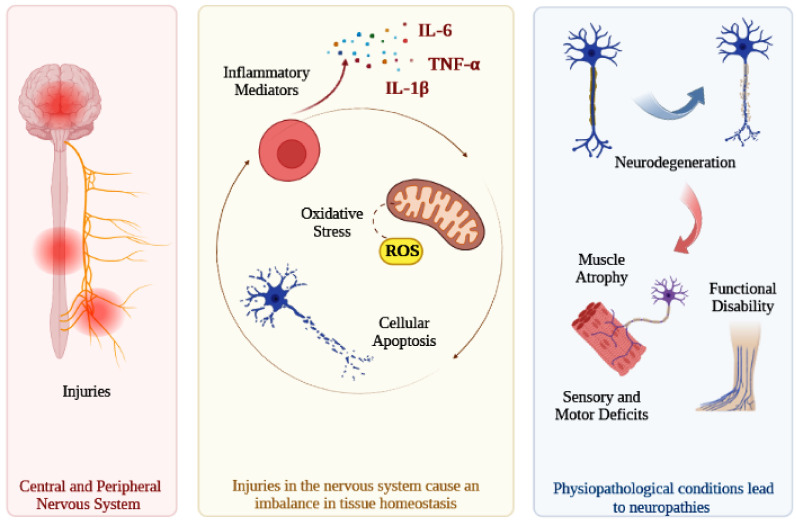
Injuries in the central or peripheral nervous system can lead to an increase in the synthesis of inflammatory cytokines, in the production of reactive oxygen species, and in cell apoptosis. These physiopathological alterations favor the degeneration of nervous cells, impair tissue regeneration, and compromise the recovery of neurological functions. Consequently, lesions in nervous tissue can cause complications of different degrees of severity, such as muscle atrophy, sensory and motor deficits, partial or total functional disability, and neuropathic pain. Interleukin-6 (IL-6); Tumor Necrosis Factor alpha (TNF-α); Interleukin-1 beta (IL-1β); Reactive Oxygen Species (ROS).

In this regard, nerve regeneration and the recovery of neurological functions are influenced by events resulting from the imbalance in the homeostasis of the injured tissue [1]. Injuries in the nervous system cause several physiopathological changes in the microenvironment that impair the regenerative process, such as increased cellular oxidative stress and elevated synthesis of pro-inflammatory cytokines, like interleukin-1 (IL-1), interleukin- 6 (IL-6), and tumor necrosis factor alpha (TNF-α) [8]. Increased concentrations of these cytokines activate the expression of other factors, such as cyclooxygenase-2 (COX-2) and inducible nitric oxide synthase (iNOS). Additionally, abnormalities that lead to mitochondrial dysfunction also promote increased oxidative stress, predisposing to alterations in neurotransmitters and neuronal activity [9]. Oxidative stress results from the accumulation of reactive oxygen species (ROS) or reactive nitrogen species (RNS) in cells and constitutes one of the main factors associated with neurodegenerative disorders [9,10,11]. The excessive production of free radicals can lead to alterations in macromolecules, such as DNA, proteins, and lipids, causing significant cellular damage and leading to cell death [9,12]. Therefore, an exacerbated and prolonged inflammatory response, increased oxidative stress, and cellular apoptosis are the main events associated with the neurodegeneration process [8]. These events favor axonal degeneration and demyelination, while inducing necrosis and apoptosis of nervous cells, such as neurons and oligodendrocytes. These physiopathological alterations can also favor the formation of a scar at the injury site, which is another factor that impairs axonal growth and tissue regeneration (Figure 1) [1].

Thus, considering the challenges encountered to obtain adequate regeneration and a satisfactory recovery of functional capacity, therapeutic strategies have been searched that can act as adjuvants to the resources available for the treatment of injured nervous tissue [3]. Therefore, the use of some bioactive agents with therapeutic potential, such as flavonoids, has been investigated as a strategy to promote tissue regeneration. Flavonoids are dietary phytochemicals, from the polyphenol class, found in a variety of fruits, vegetables, and beverages, including citrus fruits, strawberries, raspberries, apples, grapes, cocoa, legumes, grains, coffee, green tea, and red wine [13]. According to their chemical characteristics, flavonoids are classified into subgroups, such as flavonols, flavones, isoflavones, flavanones, flavanols, and anthocyanidins [14]. In general, flavonoid subgroups have nutritional properties and therapeutic potential on different pathologies, such as cancer, cardiovascular, neurological, inflammatory, and metabolic diseases [14,15,16]. Due to their antioxidant, anti-inflammatory, antiallergic, antimicrobial, antitumor, and antiviral properties, flavonoids can exert significant beneficial effect, modulating various biological processes [17]. Flavonoids exhibit the ability to scavenge ROS, activate antioxidant enzymes and inhibit enzymes related to the production of free radicals, as well as downregulate the expression and synthesis of factors related to oxidative stress, such as iNOS and nitric oxide (NO) [18,19]. Thus, the main mechanisms of action by which flavonoids exert their effect are related to their ability to inhibit ROS production and reduce the synthesis of inflammatory mediators, such as TNF-α, IL-6, interleukin-1 beta (IL-1β), COX-2, and prostaglandin E2 (PGE2) [16,20].

Considering the nutritional value and therapeutic potential of flavonoids, and with diet as a source of a wide variety of phytochemicals, studies have investigated the effect of the use of these bioactive agents in the control and progression of several pathologies. Among the various types of dietary flavonoids, quercetin is a flavonoid that is highlighted for having neuroprotective properties and biological effect on nervous tissue [10]. Thus, this literature review compiled in vivo studies that investigated the effect of quercetin administration on regeneration and recovery of functional capacity in spinal cord injury (SCI) or peripheral nerve injury models.

## 2. Flavonoid Quercetin

### Biological Properties

The term quercetin is derived from the Latin “*quercetum*”, commonly referred to as “oak forest” [12,21]. Quercetin is a bioactive agent that is widely distributed among a diversity of vegetable species and medicinal plants, such as *Ginkgo biloba, Hypericum perforatum,* and *Sambucus canadensis* [21]. Structurally, quercetin (3,3′,4′,5,7-pentahydroxyflavone) has two benzene rings linked by a heterocyclic pyran or pyrone ring and five hydroxyl groups [22], with the molecular formula C_15_H_10_O_7_ [9,12,21]. Considering the chemical aspect, quercetin has a greenish-yellow crystalline solid appearance, being responsible for the pigmentation of various fruits, flowers, and vegetables [12,23,24]. Quercetin has an important nutritional value, constituting one of the most abundant flavonoids in the diet [21,22,23,24]. The main dietary sources of quercetin are onions and apples, in addition to other foods, such as cherries, grapes, blueberries, citrus fruits, red leaf lettuce, cabbage, broccoli, tomatoes, peppers, asparagus, wine, and tea [21,22,25,26].

In fruits and vegetables, quercetin is usually present in the form of glycosides, conjugated to carbohydrate residues, such as glucose and rutinose [12,22,25,27]. After ingestion, quercetin glycosides are hydrolyzed by β-glycosidases in the intestine. Most of the aglycone form is absorbed in the gastrointestinal tract and metabolized in the liver [9,12,21,22,25]. Thus, the microbial of the gastrointestinal tract plays an important role in the degradation and metabolism of this bioactive agent [22]. Quercetin, therefore, has a rapid and extensive metabolism, being efficiently eliminated by the intestine and kidneys [9,19]. One of the important issues in this process refers to its bioavailability. In addition to the intestinal flora, some factors, such as diet, can alter the bioavailability of quercetin and its metabolites. Certain dietary elements can interfere to increase the plasmatic concentration of these molecules. Quercetin, however, has a short half-life and relatively low bioavailability, which may influence its biological effect [9,12,23,25]. Considering these issues, some studies have investigated different delivery systems that could increase the bioavailability and facilitate the access of quercetin in the target tissues, such as the use of loaded quercetin in hydrogels, nanoparticles, nanofibers, polymeric micelles, or mucoadhesive nanoemulsions [9,28,29,30,31,32]. Thus, research has advanced in the use of these technologies since the therapeutic effect of a bioactive agent depends largely on its bioavailability.

**Figure 2:**
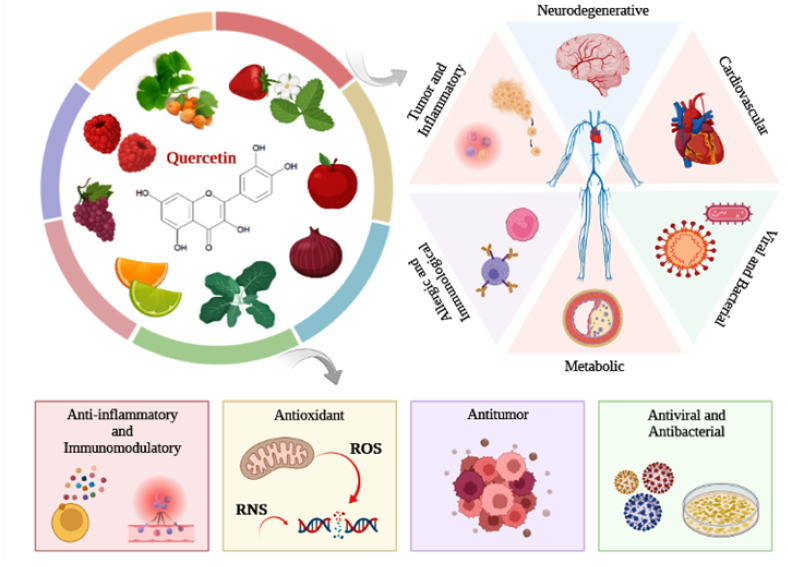
Quercetin is a dietary flavonoid widely distributed among a diversity of fruits, vegetables, and medicinal plants. Quercetin has anti-inflammatory, antioxidant, antitumor, antiviral, antibacterial, and neuroprotective properties. Due to its properties, quercetin can exert beneficial biological activity, acting in the prevention of various pathologies, such as cancer, bacterial and viral infections, cardiovascular, neurodegenerative, inflammatory, immunological, and metabolic diseases. Reactive Nitrogen Species (RNS).

As with the other flavonoids, quercetin has several biological properties that are responsible for its therapeutic potential. Quercetin has anti-inflammatory, antioxidant, anticancer, neuroprotective, immunoprotective, antiviral, and antibacterial properties [21,22,33,34,35,36]. Studies report that quercetin can act in the prevention of several pathologies, such as cancer, bacterial and viral infections, cardiovascular, neurodegenerative, inflammatory, immunological, and metabolic diseases, like asthma and diabetes mellitus (Figure 2) [12,19,21,22,24,36]. Therefore, regular consumption of a diet rich in quercetin may provide health benefits, contributing to the prevention of diseases related to aging and lifestyle [12,27]. Thus, considering that quercetin is already part of the diet and considering the scientific evidence from in vivo studies and clinical trials that did not indicate adverse toxicological effects, in 2010, high purity quercetin was recognized by the Food and Drug Administration (FDA) as GRAS (Generally Recognized as Safe) for use as a food ingredient [22]. In addition to its recognized nutritional properties, the effects of quercetin as a therapeutic agent have also been investigated in various pathological conditions, such as in the rehabilitation of neurological functions.

There is evidence in the literature that quercetin has a neuroprotective effect and the potential to favor neurogenesis and regeneration of nervous tissue [9,12]. One of the characteristics that contributes to the neuroprotective action of quercetin concerns its solubility. Despite being relatively insoluble in water, quercetin is lipophilic [9,12,21]. The lipophilic nature of quercetin facilitates its passage through the blood-brain barrier. Then, quercetin absorbed and available in the plasma can easily access the brain tissue to exert its biological activity [10,12]. In the nervous system, quercetin can act in injured areas to minimize or reverse the dysfunctions resulting from neurodegenerative disorders, as well as to delay the advance of neurological alterations [9,12]. The neuroprotective effect of quercetin is mainly related to its anti-inflammatory and antioxidant potential, since quercetin acts by protecting the tissue against oxidative stress induced or resulting from physiological metabolism [10]. In addition to physiopathological changes, physiological conditions, such as aging, can compromise the antioxidant capacity of the tissue, resulting in increased oxidative damage [9]. Overall, the antioxidant action of quercetin occurs through several mechanisms, such as free radical scavenging, chelating action on metal ions, acting on mitochondrial function, on gene expression and on the synthesis of antioxidant factors [10]. In addition to reducing ROS formation and lipid peroxidation, quercetin also acts by modulating the inflammatory response, inhibiting the synthesis of pro-inflammatory cytokines, such as TNF-α, IL-1β, and IL-6, and favoring the synthesis of anti-inflammatory cytokines, such as interleukin-10 (IL-10) [19].

The anti-inflammatory and antioxidant properties of quercetin have been reported in several studies. In vitro studies showed that quercetin reduced the production of NO and ROS, inhibited the activation of nuclear factor-kappa B (NF-kB), and downregulated the expression of inflammatory mediators, such as IL-1β, IL-6, TNF-α, and COX-2, even in lipopolysaccharide-stimulated cells (LPS) [37,38,39,40,41]. The neuroprotective properties of quercetin have also been reported in studies with animal models subjected to neuronal injury induced by trauma, hypoxia, or LPS. In animals with traumatic brain injury, quercetin administration reduced inflammatory response, oxidative stress, neuronal apoptosis, and brain edema [42,43], improving cognitive functions, biogenesis, and mitochondrial function [43,44,45,46]. Quercetin treatment also modulated the inflammatory response, minimized oxidative stress, and reduced neuronal apoptosis in animals with cerebral ischemia, suppressing the expression and synthesis of inflammatory cytokines (TNF-α, Il-1β, and Il-6), as well as inhibiting NF-kB activation [41,47]. In addition to modulating tissue responses induced by hypoxia, quercetin inhibited blood-brain barrier disruption and cerebral infarction, attenuating the neurological deficit [47].

In addition, under conditions of cerebral hypoxia, a synergistic pharmacological effect was obtained by the association of quercetin administration with the transplantation of human umbilical cord mesenchymal stromal cells (HUMSCs) [48]. In this study, treatment with quercetin and HUMSCs reduced cellular apoptosis and the synthesis of inflammatory mediators (IL-1β and IL-6), while favoring the synthesis of anti-inflammatory cytokines (IL-4, IL-10, and TGF-β1). Additionally, the combined treatment favored the survival of HUMSCs at the site of injury and promoted an improvement in the recovery of neurological functions [48]. In LPS-induced animal models, quercetin administration reduced ROS production and the synthesis of inflammatory mediators (Il-1β, TNF-α, and COX-2), minimizing neurotoxicity and neurodegeneration, in addition to improving memory function [38,49]. Similar results were obtained in studies that used quercetin to treat animals with neurodegenerative or metabolic diseases. In rotenone-induced parkinsonian rats, quercetin minimized neurological deficits and downregulated the expression of inflammatory mediators, such as Il-1β, TNF-α, and NF-kB [50]. Similarly, an inhibition of inflammatory mediator synthesis (Il-1β and TNF-α) was obtained in diabetic peripheral neuropathy animal model treated with quercetin [51]. Other in vivo studies have also reported that the administration of quercetin promoted a beneficial effect by alleviating neuropathic pain [52,53,54,55].

In general, in several studies conducted with animal models, quercetin has shown beneficial effects on the microenvironment of nervous tissue, modulating the inflammatory response, and minimizing oxidative stress, cell apoptosis and neurodegenerative disorders, in addition to alleviating neuropathic pain.

## 3. Therapeutic Effect of Quercetin

### 3.1. Animal Models with Central and Peripheral Nervous System Injuries

The influence of the neuroprotective properties of quercetin on the regeneration and functional recovery of the nervous system after injuries or traumas has also been reported in several studies. This review selected studies that investigated the effect of quercetin administration in animal models of spinal cord injury (SCI) or peripheral nerve injury and, for the most part, investigated the effect of quercetin used alone, without association with other agents [56,57,58,59,60,61]. Two studies compared the effect of administration of quercetin alone or in combination with other agent or with stem cells [62,63]. Some studies also compared the effect of quercetin administration in relation to the effect of other agents with pharmacological action, such as methylprednisolone, SB203580 (p38 mitogen-activated protein kinases inhibitor), and nerve growth factor (NGF) [64,65].

In general, the effects of the treatment with quercetin were measured using different methods of analysis, such as behavioral and electrophysiological assessment, quantitative real-time reverse-transcriptase polymerase chain reaction (qRT-PCR), Western blot, immunohistochemistry, immunofluorescence, histological assays, and motor nerve conduction velocity analysis. The main outcomes of these studies showed that quercetin favored the regeneration and recovery of the functional capacity. Table 1 synthesizes the experimental design and summarizes the main outcomes of studies that evaluated the effect of quercetin administration in animal models with spinal cord injury (SCI) or peripheral nerve injury.

#### 3.1.1. Spinal Cord Injury (SCI)

In the studies of this review that investigated the effect of quercetin administration in SCI animal models, the behavioral assessment was performed using the Basso, Beattie, Bresnahan Locomotor Rating Scale (BBB scores), which is a valid measure to detect alterations in locomotor performance after SCI [66]. In SCI animals, quercetin administration reduced histopathological damage, inflammatory cytokine synthesis, and cellular oxidative stress, promoting a significant recovery of neurophysiological functions and locomotor capacity.

Several biological conditions resulting from spinal cord injuries can influence the recovery of neurophysiological functions, such as the inflammatory process and oxidative stress, which can cause secondary damage in the injury area. Considering these issues, studies have investigated whether the use of an agent with potential to modulate the inflammatory process and oxidative stress could constitute a strategy to favor the regeneration of nervous tissue. Song et al., (2013) evaluated the effect of quercetin on cellular oxidative stress resulting from acute spinal cord injury in rats, which were treated with quercetin (0.2 mg/kg/day), methylprednisolone (30 mg/kg/day), or SB203580 (10 mg/kg/day) [65]. Methylprednisolone has been used as a neuroprotective agent in the treatment of central nervous system injuries. SB203580 is a specific inhibitor of p38 mitogen-activated protein kinases (p38MAPK) signaling. The results showed that quercetin administration significantly downregulated the expression of phosphorylated p38MAPK and iNOS, similarly to SB203580, contributing to minimize cellular oxidative stress. In contrast, the expression of these factors was significantly increased in the SCI group.

These authors also evaluated malondialdehyde (MDA) content and superoxide dismutase (SOD) activity, which are biomarkers related to oxidative stress. MDA constitutes an end product of lipid peroxidation and acts in the activation of pro-inflammatory cytokines. SOD is an antioxidant enzyme responsible for scavenging free radicals [67,68]. Quercetin treatment significantly reduced MDA content and increased SOD activity compared to SB203580. In this study, BBB scores showed that quercetin significantly improved the functional capacity of SCI animals, similarly to the positive control methylprednisolone. These authors concluded that the inhibition of p38MAPK/iNOS signaling may constitute one of the mechanisms of neuroprotective action of quercetin [65]. Corroborating with these data, Jiang et al., (2016) reported that the administration of quercetin (100 mg/kg) promoted a significant improvement in the functional recovery of SCI rats from the 3rd postoperative day, according to BBB scores [57]. SCI caused an increase in the formation of reactive oxygen species, in the production of TNF-α, and in the synthesis of pro-inflammatory cytokines, such as IL-1β and interleukin-18 (IL-18), which were significantly reduced by quercetin. Additionally, quercetin minimized histopathological damage, providing a considerable reduction in congestion, edema, neutrophil infiltration, and structural disruption in the lesion area [57].

The functional recovery of nervous tissue can also be favored by treatment with quercetin and cells with regenerative potential. Wang et al., (2018) evaluated the effect of quercetin administration (50 μmol/kg) associated with transplantation of human umbilical cord mesenchymal stromal cells (HUMSCs) in SCI rats [62]. The behavioral assessment measured by BBB scores showed that the neurological functions of the animals were significantly improved by the treatment with quercetin and HUMSCs, differing significantly from the other groups. Furthermore, the treatment combining quercetin and HUMSCs reduced the formation of cystic cavities, exhibited greater axonal preservation, and showed a significant reduction in the levels of pro-inflammatory cytokines, such as IL-1β and IL-6, in addition to exhibiting an increase in the levels of anti-inflammatory cytokines, such as IL-10, interleukin-4 (IL-4), and transforming growth factor beta 1 (TGF-β1). These authors concluded that the administration of quercetin combined with the transplantation of HUMSCs had a synergistic effect, which may constitute a strategy to favor the recovery of neurological functions and to minimize the damage resulting from SCI [62].

Some studies with SCI animal models have investigated the effect of quercetin on nerve cells, such as astrocytes and oligodendrocytes. Wang et al., (2018) evaluated the effect of quercetin (20 mg/kg/day) on astrocyte activation and on the expression of factors, such as glial fibrillary acidic protein (Gfap) and S100 calcium binding protein B (S100b) [61]. Gfap and S100b are biomarkers related to neurological damage. S100b constitutes a neurotrophic protein present in astrocytes [69]. Gfap, in turn, is the main component of the cytoskeleton of astrocytes and constitutes a marker of differentiation and activation of these cells [70,71]. Analyses showed that the quercetin-treated group showed an overexpression of Gfap and S100b at 7 days post-SCI, evidencing a positive effect on astrocyte activation. Astrocytes perform several functions in nervous tissue, being responsible for neuronal homeostasis [72]. In the early stages of the regenerative process, activation of astrocytes can be considered beneficial. However, prolonged activation of astrocytes can lead to tissue damage, making it difficult to regenerate the injured area [72]. Additionally, histological analyses also showed that quercetin considerably reduced the area of cavities at the site of injury and favored axonal regeneration, minimizing tissue damage, and promoting an increase in axon density. Thus, quercetin treatment promoted a significant improvement in functional recovery and electrophysiological capacity of SCI rats [61].

Fan et al., (2019) investigated the effect of the anti-inflammatory properties of quercetin (7.5 mg/kg) on oligodendrocyte necroptosis after SCI [56]. Oligodendrocytes are present in the central nervous system, and they act in the formation of the myelin sheath [73]. Necroptosis of oligodendrocytes is often involved with the inflammatory process, and it can be exacerbated after SCI, leading to more severe neurological damage. In this study, quercetin administration improved the functional recovery of the animals, significantly reducing myelin and axonal loss, which were markedly expressive in the SCI group. Immunofluorescence staining showed that quercetin minimized the reduction of myelin basic protein (Mbp) and neurofilament (NF200) in the white matter of SCI mice. Mbp constitutes the main component of the myelin sheath of oligodendrocytes. NF200 is a structural constituent of the cytoskeleton, and it is involved in axon development. Analysis by qRT-PCR also showed that the mRNA expression of factors involved in the inflammatory process, such as *TNF-α* and *iNOS*, were significantly downregulated by quercetin. Additionally, according to immunohistochemistry, quercetin favored the survival of oligodendrocytes by approximately 80%, significantly reducing the necroptosis of these cells after SCI [56].

The effect of quercetin administration (20 mg/kg/day), alone or associated with a specific autophagy inhibitor (3-methyladenine, 3-MA, 400 nmol) was investigated by Wang et al., (2021) [63]. BBB scores were employed over time to assess locomotor performance and recovery of functional capacity in SCI rats, with assessments performed at 1-, 3-, 7-, and 14-days post-SCI. The results showed that there was no significant difference between the scores of the three groups (SCI, SCI + quercetin, SCI + quercetin + 3-MA) on day 1. However, quercetin administration promoted a significant recovery of locomotor capacity from the 3rd day, which increased over time, differing from the SCI group. In addition, the SCI + quercetin group showed better recovery of functional capacity than the SCI + quercetin + 3-MA group, but with no significant difference between them. Similarly, treatment with quercetin favored the electrophysiological recovery of the animals, while 3-MA partially reduced the beneficial effects of quercetin. In this study, histological analysis showed that SCI caused the disruption of nerve fibers, leading to cavity formation. However, quercetin administration reduced the deformity and the degree of histological alterations in the nervous tissue, so that the lesion area and the presence of cavities were reduced in the SCI + quercetin group.

However, 3-MA partially minimized the effects of quercetin. Additional analyses performed at 14 days post-SCI showed that SCI led to other significant changes in the nervous tissue. Immunofluorescence staining showed that the SCI group had a reduction of 5-hydroxytryptamine (5-HT) or serotonin positive nerve fibers and neurofilament (NF200) positive neurons, in addition to an increase in the detection of Gfap-positive astrocytes. Quercetin administration improved this condition, promoting an increase in 5-HT positive nerve fibers and NF200 positive neurons and a decrease in Gfap-positive astrocytes, which is favorable for tissue regeneration at this more advanced stage. Quercetin also downregulated *Gfap* expression and upregulated the expression of RNA binding fox-1 homolog 3 (*Neun*), confirmed in this study by Western blotting. Immunohistochemistry staining also showed a high number of Gfap-positive cells and a reduced number of NF200 and Neun-positive cells in the SCI group. Quercetin treatment reduced Gfap-positive cells and increased NF-200 and Neun-positive cells; however, these effects were also partially inhibited by 3-MA. Another important parameter investigated in this study was cellular autophagy, evaluated by the expression of the biomarkers Beclin 1 (Beclin 1) and microtubule-associated protein 1 light chain 3 alpha (LC3-II). Autophagy is essential for neuronal homeostasis. The SCI group showed an overexpression of Beclin 1 and LC3-II in relation to Sham, on days 1 to 3 post-SCI. Quercetin upregulated the expression of these biomarkers and 3-MA partially minimized this effect. According to the data from this study, quercetin favored autophagy, which contributed to minimize neuronal damage and to improve recovery of functional capacity after SCI [63].

#### 3.1.2. Peripheral Nerve Injury

The studies of this review that investigated the effect of quercetin on the regeneration and functional recovery of the peripheral nervous system were conducted with animal models with sciatic nerve injuries. In these animal models, quercetin administration also modulated the inflammatory response and significantly inhibited oxidative stress and cellular apoptosis, favoring nerve fiber remyelination and improving sensory and motor recovery. Wang et al., (2011) implanted a silicone rubber nerve chamber filled with quercetin or saline solutions into the sciatic nerve gaps (15 mm) of rats [60]. After 8 weeks, morphometric analysis showed that the groups treated with different dosages of quercetin (0.1, 1, and 10 µg/mL) showed an adequate reinnervation of the gastrocnemius muscle and a considerable increase in the number and density of myelinated axons compared to the control.

However, at this stage, the gastrocnemius muscle still showed significant atrophy, exhibiting relatively smaller muscle fibers at the injury site. Among the experimental groups, the group treated with quercetin, at a dosage of 1 µg/mL, presented an area of evoked muscle action potential significantly greater in relation to the control. These authors also found that quercetin, at all concentrations (0.1, 1, and 10 µg/mL), significantly favored the survival and growth of Schwann cells in vitro, not inducing cellular apoptosis [60]. Chen et al., (2017) also reported that quercetin administration promoted functional recovery after sciatic nerve crush injury in mice [64]. In this study, animals were treated with quercetin, at different dosages (0.2, 2, and 20 mg/kg/day), or with mice-derived nerve growth factor (m-NGF, 4.86 µg/kg/day). Sciatic nerve crush injury caused a significant deficit in the number of myelinated fibers.

Therefore, analyses showed that quercetin (mainly at 20 mg/kg/day) and mNGF favored the expression of genes related to intrinsic axon growth and promoted an increase in the number of myelinated fibers. Intrinsic neuronal growth was evaluated by the expression of cyclic adenosine monophosphate (cAMP) and growth associated protein 43 (Gap43). In all the evaluated periods (7, 14, and 35 days), quercetin at the highest dose tested (20 mg/kg/day) showed the best results, upregulating the expression of these two factors. At 20 mg/kg/day, quercetin also significantly accelerated sensory and motor function recovery, exhibiting sensory responses more rapid than the m-NGF group. In addition, quercetin (20 mg/kg/day) and mNGF significantly reduced muscle atrophy [64].

Corroborating with these studies, Turedi et al., (2018) investigated the effects of quercetin in a rat sciatic nerve crush injury model and obtained promising results [59]. In this study, untreated (T) or quercetin (Q) treated animals (200 mg/kg/day) were sacrificed at 7 or 28 days. Morphometric analyses were performed considering several parameters, such as the thickness of the myelin sheath, the diameter of the nerve fibers and the number of myelinated nerve fibers in the injured sciatic nerve. Analyses showed that the trauma caused axonal edema, in addition to degeneration in most of the myelinated axons and in the myelin sheath, signs indicative of Wallerian degeneration. The thickness of the myelin sheath and the number of myelinated nerve fibers were significantly compromised by the injury to the sciatic nerve, so that the T-7 and T-28 groups had a lower number of myelinated nerve fibers, with a thinner myelin sheath, compared to the Sham groups (S-7 and S-28).

Regarding the evaluated periods, analyses showed that the T-7 group presented a more expressive degree of nerve fiber degeneration; however, the T-28 group already exhibited the presence of nerve fibers indicating a process of initiated regeneration. Comparing the treated and untreated groups, analyses showed that quercetin favored and accelerated the recovery of the injured nerve, since the T + Q-28 group showed a more advanced degree of regeneration compared to the T-28 group, exhibiting visible histopathological findings. Thus, T + Q-28 presented a greater number of myelinated nerve fibers, showing thicker myelin sheaths compared to T-28, with significant differences between these groups. Considering quercetin treatment and experimental periods, analyses showed that quercetin promoted an improvement in morphological parameters over time, since the T + Q-28 group had a significantly greater number of myelinated nerve fibers with thicker myelin sheaths in relation to the group treated for only 7 days (T + Q-7).

Therefore, the regeneration of the nervous fiber structure was significantly more expressive in the T + Q-28 group compared to the T + Q-7 group, which indicates that the regeneration process advanced over the days. These authors also performed biochemical analyses to evaluate the apoptosis index among the Schwann cells of the sciatic nerve. The data from these analyses showed that injury to the sciatic nerve increased the rate of cell apoptosis, as this rate was significantly higher for the T-7 and T-28 groups compared to the Sham groups (S-7 and S-28). In animals submitted to trauma, the administration of quercetin significantly reduced the apoptosis index, which was evident in the comparison of T + Q-7 in relation to T-7, as well as between T + Q-28 and T-28, with no increase in this index over time for all groups. Considering the results of the analyses, the authors concluded that quercetin had beneficial effects and potential to shorten the period of nerve regeneration [59].

Qiu et al., (2019) evaluated the effect of isoquercitrin (quercetin-3-glucoside), at 20 mg/kg/day, in mice sciatic nerve crush injury model [58]. Sciatic nerve injury resulted in a significant reduction in the thickness and number of layers of the myelin sheath, in addition to a reduction in the expression of proteins involved in the formation and conservation of the myelin sheath, such as myelin-associated glycoprotein (Mag) and peripheral myelin protein 22 (Pmp22). Mag is expressed by oligodendrocytes of the central nervous system and by Schwann cells present in the peripheral nervous system. Pmp22 is involved in several functions, including the formation and conservation of the myelin sheath of cells in the peripheral nervous system. Analyses performed at 15 days post-injury showed that the administration of isoquercitrin reversed part of these events, upregulating the expression of Mag and Pmp22, as well as the expression of factors involved with axonal growth, such as Gap43 and NF200. Isoquercitrin showed beneficial effects, favoring peripheral nerve remyelination, improving motor function recovery, and reducing muscle atrophy.

Additionally, isoquercitrin suppressed cellular oxidative stress resulting from sciatic nerve injury, downregulating the expression of proteins related to ROS production, such as NADPH oxidase 4 (Nox4) and dual oxidase 1 (Duox1), and upregulating the expression of proteins involved with the inhibition of free radical production, such as nuclear factor erythroid 2-related factor-2 (Nrf2) and superoxide dismutase 2 (Sod2). In this study, in vitro assays also showed that isoquercitrin promoted the proliferation and migration of Schwann cells in a dose-dependent manner. These authors concluded that isoquercitrin may have considerable therapeutic potential as a neuroprotective agent [58].

**Figure 3:**
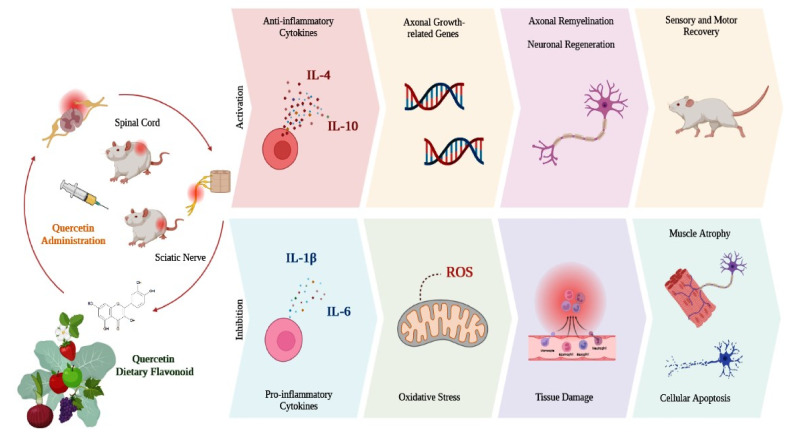
Schematic illustration of the main outcomes of the studies included in this literature review. In SCI and sciatic nerve injury models, quercetin administration inhibited the synthesis of pro-inflammatory cytokines, oxidative stress, and cell apoptosis, thus minimizing tissue damage and muscle atrophy. Similarly, quercetin favored the synthesis of anti-inflammatory cytokines and the expression of growth-related genes, promoting axonal remyelination and neuronal regeneration, accelerating sensory and motor recovery, and improving locomotor capacity. Interleukin-4 (IL-4); Interleukin-10 (IL-10); Interleukin-1 beta (IL-1β); Interleukin-6 (IL-6); Reactive Oxygen Species (ROS).

Figure 3 illustrates and summarizes the main outcomes of the in vivo studies that were included in this literature review, considering the effects of quercetin administration in animal models with SCI or sciatic nerve injury.

In addition to the content reported in this review, complementary therapies can also be considered, which can also be used in peripheral nerve regeneration, such as the use of Mesenchymal Stem Cells (MSCs) [74], fibrin sealants or fibrin “glues” [75], and photobiomodulation therapy (PBM), with the use of low-level laser (LLLT) [76] or light emitting diode (LED) [77], to improve the process of morphological and functional recomposition of injured nervous tissues, in which we envisage future studies to be carried out.

## 4. Conclusions

Injuries and traumas that affect the nervous system cause an imbalance in tissue homeostasis, leading to several physiopathological alterations, such as increased synthesis of inflammatory mediators, oxidative stress, and cellular apoptosis. These alterations may result in secondary damage in the injury site that can impair adequate nerve regeneration, compromising the recovery of functional capacity. The use of agents with antioxidant and anti-inflammatory properties, such as flavonoids, may constitute a strategy to favor the regeneration of nervous tissue. Quercetin is a dietary flavonoid, from the flavonols subgroup, which has neuroprotective properties and biological activity on nervous tissue. In animal models with SCI or sciatic nerve injuries, quercetin administration reduced histopathological damage, inflammatory cytokine synthesis, and cellular oxidative stress, promoting a significant recovery of neurophysiological functions and locomotor capacity. Although there is still a lack of clinical research, in vivo studies have shown that quercetin may have a beneficial effect on the nervous system, with the potential to minimize deleterious alterations, to favor regeneration and to improve the recovery of neurological functions.

## Figures and Tables

**Table 1:** In vivo studies that evaluated the effect of quercetin administration in animal models with spinal cord injury (SCI) or peripheral nerve injury (sciatic nerve).

References	Animals Models	Treatment Groups	Intervention	Main Analysis	Main Outcomes
Wang et al., (2011) [60]	Sprague Dawley rats. Sciatic Nerve Injury	G1: Saline solution G2: Quercetin 0.1 µg/mL G3: Quercetin 1 µg/mL G4: Quercetin 10 µg/mL	Implantation of silicone rubber nerve chamber filled with the quercetin or saline solutions in the gaps (15 mm) (*n* = 10). Analyses were performed after 8 weeks of the procedures.	Electrophysiological and histological analysis.	Quercetin-treated groups showed a considerable increase in the number and density of myelinated axons in relation to the control, with satisfactory reinnervation of the gastrocnemius muscle. Quercetin (1 µg/mL) had a considerably larger area of evoked muscle action potential than the control group.
Song et al., (2013) [65]	Male Sprague Dawley rats. Spinal Cord Injury (SCI)	G1: Sham surgery G2: SCI G3: SCI + Quercetin 0.2 mg/kg/day G4: SCI + Methylprednisolone (MP) 30 mg/kg/day G5: SCI + specific p38MAPK inhibitor SB20358 (SB) 10 mg/kg/day	Intraperitoneal injections of quercetin, MP, or SB solutions (*n* = 8). Analyses were performed until the 14th day after the procedures.	Behavioral assessment (BBB: Basso, Beattie and Bresnahan scores), qRT-PCR, Western blot, and immunohistochemical analysis.	Quercetin significantly improved BBB scores, similarly to the positive control (MP). Quercetin suppressed the expression of inducible nitric oxide synthase (iNOS) similarly to SB, showing a neuroprotective effect by inhibiting cellular oxidative stress.
Jiang et al., (2016) [57]	Female Sprague Dawley rats. Spinal Cord Injury (SCI)	G1: Sham G2: SCI G3: SCI + Saline solution (vehicle) G4: SCI + Quercetin solution 100 mg/Kg	Intraperitoneal injections with quercetin or vehicle solutions at 12-h intervals for 3 days (*n* = 5). Analyses were performed until the 14th day after the procedures.	Behavioral assessment (BBB scores), Western blot, histological assays, and biochemical analysis.	Quercetin promoted a significant improvement in functional recovery, reducing histopathological damage, inflammatory cytokines synthesis, and reactive oxygen species production.
Chen et al., (2017) [64]	Male C57BL/6J mice. Sciatic Nerve Crush Injury	G1: Sham G2: Saline solution G3: Quercetin 0.2 mg/kg/day G4: Quercetin 2 mg/kg/day G5: Quercetin 20 mg/kg/day G6: mice-derived nerve growth factor (mNGF) 4.86 µg/kg/day	Injection of the solutions into the plantar muscle of the left hind limb once a day (*n* = 10). Analyses were performed at 7, 14, and 35 days after the procedures.	Behavioral test, qRT-PCR, Western blot, immunofluorescence, transmission electron microscopy, and motor nerve conduction velocity analysis.	Quercetin (mainly at 20 mg/kg/day) and mNGF favored the expression of genes related to intrinsic axon growth and promoted an increase in the number of myelinated fibers. At 20 mg/kg/day, quercetin significantly accelerated sensory and motor function recovery. In addition, quercetin (20 mg/kg/day) and mNGF significantly reduced muscle atrophy.
Turedi et al., (2018) [59]	Male Sprague Dawley rats. Sciatic Nerve Crush Injury (T)	G1: Sham (S-7) G2: Sham (S-28) G3: Quercetin (Q-7) 200 mg/kg/day G4: Quercetin (Q-28) 200 mg/kg/day G5: T (T-7) G6: T (T-28) G7: T + Quercetin (T + Q-7) 200 mg/kg/day G8: T + Quercetin (T + Q-28) 200 mg/kg/day	Intragastric administration of quercetin solutions for 7 days (*n* = 6). Analyses were performed at 7 and 28 days after the procedures.	TUNEL assay, histopathological assay, and biochemical analysis.	Quercetin significantly decreased the index of apoptosis. Nerve fiber regeneration was significantly more expressive in T + Q-28 than in T + Q-7. In addition, T + Q-28 showed significantly more myelinated nerve fibers with thicker myelin sheaths than T + Q-7.
Wang et al., (2018) [62]	Female Sprague Dawley rats. Spinal Cord Injury (SCI)	G1: Sham G2: Culture medium G3: Human umbilical cord mesenchymal stromal cells (HUSMCs) G4: Quercetin 50 μmol/kg G5: HUSMCs + Quercetin 50 μmol/kg	Administration of quercetin or saline solutions at 12-h intervals for 3 days. HUSMCs transplantation (2 dosages) into the injured spinal cord. (*n* = 28). Analyses were performed until the 4th week after the procedures.	Behavioral assessment (BBB scores) and immunohistochemical analysis.	HUMSCs + Quercetin promoted significant improvement in neurological function in relation to the other groups. Similarly, HUMSCs + Quercetin reduced cystic cavities formation, inflammatory cytokines synthesis, and iNOS production, while favoring pro-inflammatory cytokines synthesis.
Wang et al., (2018) [61]	Male Sprague Dawley rats. Spinal Cord Injury (SCI)	G1: Sham G2: SCI G3: SCI + Quercetin 20 mg/kg/day	Intraperitoneal injections of quercetin for 7 days (*n* = 10). Analyses were performed after 7 days of the procedures.	Behavioral assessment (BBB scores), qRT-PCR, Western blot, immunofluorescence, histological assays, and electrophysiological analysis.	Quercetin significantly improved functional capacity and electrophysiological recovery. Quercetin reduced cavity formation, favored axonal regeneration, and promoted astrocyte activation, upregulating the expression of glial fibrillary acidic protein (GFAP) and S100 calcium binding protein B (S100β).
Fan et al., (2019) [56]	Male Sprague Dawley rats. Spinal Cord Injury (SCI)	G1: Sham G2: SCI + saline solution (vehicle) G3: SCI + Quercetin 7,5 mg/kg	Intraperitoneal injections of quercetin or vehicle solutions twice daily for 10 days (*n* = 6). Analyses were performed until the 21st day after the procedures.	Behavioral assessment (BBB scores), qRT-PCR, Western blot, immunohistochemical assays, and electron microscopic analysis.	Quercetin significantly improved functional recovery. Quercetin considerably prevented oligodendrocyte necropsies, in addition to significantly reducing myelin loss and axonal loss after SCI.
Qiu et al., (2019) [58]	Male ICR mice. Sciatic Nerve Crush Injury	G1: Sham G2: Saline solution (vehicle) G3: Isoquercitrin (quercetin-3-glucoside) 20 mg/kg/day	Intraperitoneal injections of isoquercitrin or vehicle solutions. Analyses were performed until the 23rd day after the procedures.	Behavioral assessment (sciatic functional index), cell proliferation and migration assays qRT-PCR, Western blot, and electrophysiological analysis.	Isoquercitrin favored peripheral nerve remyelination, improved motor function recovery, reduced muscle atrophy, and inhibited autophagy. In addition, isoquercitrin suppressed cellular oxidative stress, favoring the proliferation and migration of Schwann cells.
Wang et al., (2021) [63]	Male Sprague Dawley rats. Spinal Cord Injury (SCI)	G1: Sham surgery + saline solution G2: SCI + saline solution G3: SCI + Quercetin 20 mg/kg/day G4: SCI + Quercetin + 3-methyladenine (3-MA; 400 nmol)	Intraperitoneal injections of solutions for 1, 3, or 7 days (*n* = 10). Analyses were performed until the 14th days after the procedures.	Behavioral assessment (BBB scores), Western blot and immunohistochemical analysis.	Quercetin favored axonal regeneration and promoted a significant recovery of locomotor capacity, minimizing histological alterations and cavity formation. 3-MA partially abrogated the neuroprotective effects of quercetin.
